# The Impact of Core Self-Evaluations on Job Satisfaction and Turnover Intention among Higher Education Academic Staff: Mediating Roles of Intrinsic and Extrinsic Motivation

**DOI:** 10.3390/bs12070236

**Published:** 2022-07-15

**Authors:** Abisola Leah Akosile, Mehmet Ali Ekemen

**Affiliations:** Department of Business, Faculty of Economics and Administrative Sciences, European University of Lefke, Lefke 99770, Northern Cyprus, Turkey; mekemen@eul.edu.tr

**Keywords:** core self-evaluations, job satisfaction, turnover intention, intrinsic motivation, extrinsic motivation, academic staff

## Abstract

Job satisfaction and turnover intention among academic staff remains a challenge in higher education institutions. To aid understanding of the factors that can reduce intention to leave and increase job satisfaction among academic staff, the present research investigated the impact of core self-evaluations (CSEs) on job satisfaction and turnover intention by proposing a parallel mediation model. The researcher used quantitative approach. The sample consisted of (*n* = 305) academic staff working in higher education institutions in Nigeria, with a total of 80 females and 225 males. The study attempted to investigate the connection between core self-evaluations, job satisfaction, and turnover intention using self-determination theory to investigate the parallel mediating role of intrinsic and extrinsic motivation on the relationship. Through application of structural equation modeling, the findings showed that CSEs had an impact on job satisfaction and turnover intention, mainly through the mediating role of intrinsic and extrinsic motivation. The mediating role of intrinsic and extrinsic motivation provided new insight into the connections between core self-evaluations, job satisfaction, and turnover expectations.

## 1. Introduction

Higher education is an exceptionally complicated system [1,2]. Its complexity results from the necessity for teaching and learning activities (involving collaboration, research, and several other factors) to result in high-quality learning outcomes and improve the quality of education systems globally [1,3]. The continued increasing demand for higher education participation, the rankings of higher education institutions (HEIs), and the competition among the world’s HEIs have added further complexity to the higher education system. HEI management must thus develop appropriate strategies to meet the growing demand and to guarantee quality assurance and learning outcomes [4,5]. However, achieving high-quality learning outcomes, innovative research, and an improved education system is unattainable without the academic staff, which makes them important assets of HEIs [6,7,8]. The work [6] demonstrated that academic staff contribute to institutional visibility, scientific advancement, and economic activity through teaching and innovation. As academic staff are the major contributors to the success of HEIs, understanding what motivates them is very essential.

Furthermore, there is an abundance of evidence that suggests academic staff are leaving their jobs at a very high rate compared to other jobs [9]. Hence, for executives, it is important to find ways to appeal to and recruit academic staff, and then be able to ensure they are retained in the education sector [10]. According to [11] job satisfaction and motivation among academic staff is a vital contributing factor to the positive results of HEIs.

A meta-analytic finding showed that personality trait can explain a certain percentage of variance in wellbeing measures, for example, burnout and work engagement. In recent times, significant focus has been placed on personal differences because they have been connected to positive job outcomes such as job motivation. There is a necessity to understand how personality traits contribute to staff wellbeing [12]. In this respect, understanding the personality trait that impacts the motivation of academic staff is important for HEI(s). In the present study, we focused on the role of a wide personality construct, CSEs, which are an individual’s important assessment of their own pride, ability, and effectiveness [13]. Considering that CSEs has been proposed as an emotional outlook for job satisfaction and life satisfaction, research has begun in recent times to test CSEs as a personal resource and the effect it has on job satisfaction, performance, and work outcomes [14]. CSEs signifies the basic evaluation a person makes about their capabilities. CSEs are conceptualized as a higher-order construct made up of large and evaluative traits such as generalized self-efficacy, self-esteem, locus of control, and neuroticism [15].

The present study aims to contribute to this area of study in three ways. First, we tested the direct effect of CSEs on job satisfaction and turnover intention. Second, to provide a detailed description of how CSEs relate to job satisfaction and turnover intention, we used the theoretical framework of self-determination theory [16]. We expected motivation to be an important mediator in the relationship between CSEs, job satisfaction, and turnover intention due to the assumption that the relationship between CSEs and motivation in the intention of an individual may influence their perceptions of their job [17]. Third, some scholars e.g., [18,19,20,21,22] have suggested that CSEs can be an essential determinant in the improvement of job satisfaction, CSEs can have a direct relation with turnover intention [23], and that an insufficient number of studies have been conducted concerning the combined role of intrinsic and extrinsic motivation in predicting staff work output [24]. Hence, we focused on the mediating effect of intrinsic and extrinsic motivation. We tested whether intrinsic and extrinsic motivation mediates the relationship between CSEs, job satisfaction, and turnover intention.

## 2. Theoretical Framework and Hypotheses

### 2.1. Self-Determination Theory (SDT)

SDT is the difference between controlled motivation and freedom motivation. Freedom involves acting in freewill; intrinsic motivation is an example of freedom motivation, for example, when people perform a task because they find it amusing, they are doing the task completely voluntarily (e.g., I work because it is exciting). On the other hand, being controlled includes performing with a sense of pressure. In early experiments, the use of extrinsic remuneration was discovered to influence controlled motivation [25].

### 2.2. Core Self-Evaluations (CSEs)

CSEs are an individual’s important assessment of their own pride, ability, and effectiveness [13]. CSEs incorporate four broadly examined ideas, specifically locus of control, which includes internal and external locus of control (it refers to a person’s belief about what causes events in life); self-esteem (i.e., the worth one places on oneself); neuroticism (i.e., tendency to show poor emotional adaptation and negative methods of perception); and self-efficacy evaluation of an individual’s capability to handle situations in several ways [26].

CSEs have been demonstrated to be a strong indicator of a few outcomes in the workplace [12]. For instance, a person with higher CSEs was found to recognize their tasks as supporting more internal attributes, for example, questioning oneself due to performing a task incorrectly [27]. Several illustrative diagrams depicting why CSEs influence work output have been proposed [13]. Other than an immediate impact, for instance, on work fulfillment through enthusiasm for the task ahead, CSEs may impact work output in an indirect way by influencing perceptions and evaluations, or the activities conducted in a particular circumstance. Without a doubt, CSEs have been shown to impact the perception of work quality [22]. People with positive CSEs assess circumstances in an unexpected way, have a stronger sense of self-efficacy, and show a higher level of inspiration to seek promising circumstances than individuals who are less persuaded of their capacities [15]. Given this, CSEs have been shown to have potential as individual-related assets through the investigation of their immediate, circuitous, and direct impacts on wellbeing and motivation. Various methodologies have been implemented to identify the different mental, social, and underlying assets at work [12].

### 2.3. Core Self-Evaluations and Intrinsic and Extrinsic Motivation

Self-determination theory (SDT) started with a target on intrinsic motivation [28]. In SDT, we differentiate between several types of motivation based on types of aims and goals that initiate an action. Intrinsic motivation being a type of the motivation is defined as performing an activity for implicit satisfactions instead of for some distinct effect. When a person is intrinsically motivated, they act for the challenge or pleasure instead of because of rewards or pressures. On the other hand, “Extrinsic motivation is a construct that pertains whenever an activity is done in order to attain some separable outcome” [29].

Self-determination theory focuses on the differentiation between freedom motivation and restrained motivation [30]. Freedom motivation includes performing with a sense of free will and having the knowledge of the best option. Intrinsic motivation is a symbol of freedom motivation. For example, when a person has a special interest in something, they perform that task with complete willingness [25]. Furthermore, a person with higher CSEs recognizes their tasks as supporting more internal attributes [27]. In accordance with this assumption, many research studies have stated that there is a clear connection between CSEs and motivation, which may impact individual employees’ attitudes towards their work tasks [17]. For instance, ref. [31] discovered that the mediation of intrinsic motivation on the relationship between customer incivility and staff service performance varied based on staff CSEs.

In the career world of employees, motivation is the incentive of their skillful action from start to finish, as well as a necessary determinant in the growth of an organization and person. For a while now, scholars have explored all areas of motivation. Several research have confirmed that intrinsic and extrinsic motivation have a strong effect on staff actions and character, for instance, according to [23] “the findings suggest that the key to improve employee engagement relies on maintaining their intrinsic motivation”. A person that is intrinsically motivated is likely to be compelled by basic interest in the job and pleasure, so that they feel motivated to do their job [32]. In addition, a person’s knowledge about their abilities to do a job will affect their motivation to look for or leave a job. The greater a person’s recognized self-efficacy, the more demanding the actions which that person chooses to engage in [33].

We expected CSEs to have different impacts as an individual asset in interaction with motivation. In accordance with the foundation knowledge on the various types of CSEs in the literature pertaining to job motivation [12], we anticipated a direct connection with motivation. In addition, positive self-assessments have been demonstrated to be related to high methodology propensity score and, in this manner, result in a focus towards certain data or work circumstances [34]. In accordance with this development, CSEs have been discovered to be related to positive encounters at work, such as intrinsic inspiration [12]. We proposed the following:

**Hypothesis** **(H1a).** *Core self-evaluations have a positive significant impact on intrinsic motivation*.

**Hypothesis** **(H1b).** *Core self-evaluations have a positive significant impact on extrinsic motivation.*

### 2.4. Core Self-Evaluations and Job Satisfaction

Scholars have continuously described a significant link between CSEs and job satisfaction [35]. Organizations that take staff job satisfaction into consideration are normally regarded as successful, because when a staff member is satisfied, they sustain great work productivity, and most of the time stay with the organization. Job satisfaction includes personal accomplishments that push the staff to look forward to making new accomplishments [36]. A previous study reported CSE to be associated with job satisfaction of Germans in both predictive and concurrent research [37]. Furthermore, empirical study of the relationship between CSEs and job satisfaction discovered that the CSE measure is greatly linked to job satisfaction, and another study discovered that CSEs are completely linked with job satisfaction. Another study report showed that CSEs of workers in China are linked with job satisfaction. Hence, previous results have sufficiently shown the connection between CSEs and job satisfaction [37,38]. Furthermore, findings show that staff who encounter working conditions that raise intrinsic and extrinsic motivation are more satisfied with their jobs [39].

As proposed by self-determination theory, internal motivation is connected to personal requirement of self-reliance, in which a person wishes to feel in possession of their personal action and their ability (such as wanting to generate desired results and to have mastery of knowledge) and to feel relevant to others being satisfied [30]. We proposed the following:

**Hypothesis** **(H2).** *Core self-evaluations have a positive significant impact on job satisfaction.*

### 2.5. Core Self-Evaluations and Turnover Intention

Several definitions of turnover intention have been discovered in past pieces of literature. Turnover intention is defined as the personal evaluation a person makes regarding leaving their organization. In addition, turnover intention is defined as the contemplation made by a staff member to leave their company and in a certain period look for a new job [40].

Prior study has used self-determination theory (SDT) to understand staff exploitation and results in terms of performance, wellbeing, and motivation. According to [41], a greater need for satisfaction in mental health employees has also been shown to enhance worker motivation through positive behavior. In a situation where needs are not met, it has increased turnover intentions of staff. These outcomes showed the use of self-determination theory’s basic psychological needs such as competence, autonomy, and relatedness in comprehending mental health staff results, as the staff already have the fear of low job satisfaction and turnover intentions [42].

For decades, job satisfaction has been an important determinant in predicting turnover intention [19]. However, exploring the direct connection between core self-evaluations and turnover intention would be justifiable. In addition, a meta-analysis recognized that CSEs are negatively related to turnover intention [19]. Few current research studies have examined the connection between CSEs and turnover intention. This research proposed that CSEs are negatively related to turnover intention; a person with low CSEs shows a clear need to quit their institutions and their job [43]. Some studies have investigated the direct impact of CSEs on turnover intentions, for example, in a study of Indian retail sector CSEs, they were related negatively to turnover intentions [44]. It is expected that CSEs will relate negatively to turnover intentions. Hence, we proposed the following:

**Hypothesis** **(H3).** *Core self-evaluations are negatively related to turnover intention.*

### 2.6. Intrinsic and Extrinsic Motivation, Job Satisfaction, and Turnover Intention

For staff to obtain job satisfaction, intrinsic and extrinsic motivation are essential. Two separate studies showed that intrinsic and extrinsic motivation had a significant effect on job satisfaction [45]. Another study discovered that extrinsic motivation has a negative relation with job satisfaction; “the negative sign for extrinsic motivation and job satisfaction is a surprise” [46].

According to [47], if employees are not motivated, their intention to leave their job will increase and they will become angrier and feel worthless. Several other studies that explored job satisfaction and motivation agree with this assertion (e.g., [48,49]. Research has proposed that employees see intrinsic satisfaction as a priority [50]. However, a greater number of research studies have proposed that both intrinsic and extrinsic factors are a good predictor of job satisfaction [51].

Intrinsic determinants have been widely recognized as a necessary factor for motivation [52]. The two-factor theory of Herzberg (e.g., satisfaction and motivation) proposed the determinants of staff satisfaction are internal factors of their work surroundings, where they are committed to do their work [53]. Motivational factors can possibly increase staff work motivation and job satisfaction. A study discovered that motivation had a good predictive position in deciding satisfaction, and it was confirmed that internal motivation had significant effect on satisfaction [53]. Hence, we proposed the following:

**Hypothesis** **(H4a).** *Intrinsic motivation has a positive significant impact on job satisfaction.*

**Hypothesis** **(H4b).** *Extrinsic motivation has a negative impact on job satisfaction.*

Human resource management and organizational research has investigated the connection between turnover intention and various forms of extrinsic and intrinsic motivation. A person with less satisfaction usually has a deficiency in motivation to give their best effort to their work; this lack of motivation can initiate a rise in staff turnover, for instance, the study of Millennial and older generation workers in U.S. federal agencies, in terms of their “turnover intentions and work motivations showed that they are more likely than their older counterparts to report an intention to leave their jobs, and most work attributes do not matter more for Millennial workers’ decisions to leave” [54].

Furthermore, some research has investigated the connection among the different types of motivation and several work outcomes in organizational environments, including turnover intention. For example, intrinsic motivation was found to be negatively connected with turnover intention (e.g., [23,24,25] and extrinsic motivation was found to be positively connected to turnover intention [55]. Furthermore, using self-determination theory, the findings in public sectors revealed that intrinsic motivation had a significantly negative effect on turnover intention among local revenue officers [56].

Hence, the following hypotheses were proposed:

**Hypothesis** **(H5a).** *Intrinsic motivation has a negative significant impact on turnover intention.*

**Hypothesis** **(H5b).** *Extrinsic motivation has a negative significant impact on turnover intention.*

### 2.7. Intrinsic and Extrinsic Motivation as a Mediator

Regarding the relationship between CSEs and motivation [28] self-determination theory suggests a helpful theoretical framework for understanding why CSEs are related to motivation. Core self-evaluations have been used as an instrument to evaluate motivation, several research works have specified that a positive connection between core self-evaluations and motivation possibly will lead to the option of committing themselves to or take part in the task activities, people with high core self-evaluations are believed to hold positive self-idea that increases motivation level [18], and staff with higher CSEs can experience more job satisfaction [57]. If the level of CSEs of the staff is increased, the level of their job satisfaction will also increase [58].

In self-determination theory, it is assumed that it is important to examine motivation from a logical and unreasonable level; extrinsic and intrinsic motivation drives a person’s decision making. Extrinsic motivation implies that a person is performing a certain task to obtain a certain type of outcome, whereas intrinsic motivation implies that people perform a certain task for internal satisfaction and fulfilment [59]. Intrinsic and extrinsic motivation are factors that affect job satisfaction [36].

Self-determination theories suggest that helpful work surroundings, which inspire intrinsic motivation, will lead to increased job satisfaction [46]. According to [60], both intrinsic and extrinsic motivation help increase staff job satisfaction. For instance, self-determination theory has been specifically useful for comprehending intrinsic and extrinsic motivation [61]. Intrinsic and extrinsic motivation greatly contributes to staff job satisfaction [62].

The studies on CSEs have continually revealed necessary information as well as a good understanding of how a person’s character is connected to significant career-related ideas; one of these ideas is job satisfaction [63]. Many studies have described mediators that can explain why CSEs are linked to job satisfaction; these mediators include goal setting, performance, and motivation [64]. Furthermore, a direct impact model states that CSEs affect job satisfaction through emotional generalization, and perceived job characteristics were discovered to mediate the relationship between CSEs and job satisfaction [38].

A parallel mediation test supported self-determination theory prediction of satisfaction [61]. With the support from self-determination theory of intrinsic and extrinsic motivation, we propose that parallel mediation of intrinsic and extrinsic motivation can serve as a mediator between CSEs and job satisfaction. There are currently no specific studies testing the mediational role of the combination of intrinsic and extrinsic motivation in the relationship between CSEs and job satisfaction. With the lack of literature, we specifically explored intrinsic and extrinsic motivation as a mediator. Therefore, the following hypotheses were proposed:

**Hypothesis** **(H6a).** *The relationship between core self-evaluations and job satisfaction is mediated by intrinsic motivation*.

**Hypothesis** **(H6b).** *The relationship between core self-evaluations and job satisfaction is mediated by extrinsic motivation.*

On the other hand, many researchers have found a negative and important relation between intrinsic motivation and turnover intention [65,66], possibly because staff with appealing, inspiring, and pleasant work have low interest in leaving and because they are probably not interested in the extrinsic compensation proposed by other institutions [38]. Researchers showed that there is a negative link between staff intention to leave and intrinsic motivation. Thus, if staff are less self-interested in their work, they might have low intrinsic motivation, which will lead to low productivity and encourage them to quit their job [67].

Staff with high CSEs are certain of their capabilities and potential and are less affected by external signals. How they feel, their thoughts, and actions are unlikely to be controlled by the events of their workplace and other external determinants. Hence, they are unlikely to look for a different job; they tend to continue to work with their current company. A negative correlation between CSEs and turnover intentions was discovered [44]. Staff who evaluate themselves highly and positively will me more motivated and enduring at their workplace. They are found highly positive in their capabilities, strengths, and actions and see themselves to be successful; this confidence in them increases their performance and reduces their intention to leave [68]. Hence, the following hypotheses were proposed:

**Hypothesis** **(H7a).** *The relationship between core self-evaluations and turnover intention is mediated by intrinsic motivation*.

**Hypothesis** **(H7b).** *The relationship between core self-evaluations and turnover intention is mediated by extrinsic motivation.*

## 3. Materials and Methods

### 3.1. Participants

According to the NUC statistical digest 2017, the total population of HEIs in Nigeria at the time was 146 HEIs, with a total of 61,999 academic staff [69]. Using Yamane formula of sampling, the sample size was 397. The ongoing study took place between May 2020 and September 2020. The participants were academic staff in HEIs in Nigeria and the survey was administered online by the authors of the study. The survey was created with the online tool SurveyMonkey and the survey items were given in a random order to avoid sequencing effects in the survey. No incentives were offered to research participants. The research followed the ethical standards for psychological studies, such as anonymity and confidentiality, an introduction letter, and voluntary participation. During the period of data collection, 414 academic staff clicked on the link to the online survey, and 346 started to answer the questions; 305 provided complete data. A completion rate of 74% was recorded. Male academic staff (73.8%) constituted the majority participant group in the study.

### 3.2. Measures

#### 3.2.1. Core Self-Evaluations

CSEs were measured with 12 items adopted from [70]. Sample items included “I am sure I get the victory I merit in life” and “I don’t feel in control of my success in my career”. Members rated how well each statement represents their self-evaluations on a five-point Likert scale (e.g., 1: strongly disagree, 2: disagree, 3: neither agree nor disagree, 4: agree, 5: strongly agree). Six items were reverse-coded. For the CSE scale, [57] reported a coefficient alpha of 0.75. Within the present study, the Cronbach alpha value for the CSE scale was 0.71.

#### 3.2.2. Intrinsic and Extrinsic Motivation

A motivation scale adopted from [71] was utilized to measure participants’ motivational components. It incorporated ten items in which six items measured intrinsic motivation, such as “The errands that I do at work are themselves speaking to a driving control in my job”, and four items measured extrinsic motivation, such as “If I had been offered better pay, I would have done a better job”. Participants rated how well each article represents their self-evaluations on a five-point Likert scale (e.g., 1: strongly disagree, 2: disagree, 3: neither agree nor disagree, 4: agree, 5: strongly agree). Within the current research, the Cronbach’s alpha coefficients were 0.89 for intrinsic motivation and 0.84 for extrinsic motivation.

#### 3.2.3. Job Satisfaction

The job satisfaction instrument from [72] was utilized to survey the level of job satisfaction of participants [73]. Participants were presented with 16 items and asked to rate their level of fulfillment; 9 items measured their extrinsic work satisfaction, “the way your firm is managed”, while 7 items measured their intrinsic work fulfillment, such as “The opportunity to choose your own strategy of working”. A five-point Likert scale was used to obtain participants’ ratings (e.g., 1: very dissatisfied 2: dissatisfied, 3: neither satisfied nor dissatisfied, 4: satisfied, 5: very satisfied). Within the present research, the Cronbach’s alpha coefficient for job satisfaction was 0.70. Two observed pointers for the latent variable of work satisfaction were created.

#### 3.2.4. Turnover Intention

Turnover intention was measured using the instrument adopted from [74]. This instrument included three items, such as “I often think about quitting”, and participants were asked to indicate their agreement with the statements on a five-point Likert scale (e.g., 1: strongly disagree, 2: disagree, 3: neither agree nor disagree, 4: agree, 5: strongly agree). The Cronbach’s alpha coefficient was 0.89.

#### 3.2.5. Control Variables

The control variables in this research were gender, age, education level, and organizational tenure in years. Gender was evaluated using a female (1) and male (2) dichotomy, while education was assessed in levels: undergraduate (1), masters (2), and doctorate (3). Additionally, age and organizational tenure (in years) can affect job satisfaction [4].

### 3.3. Data Analysis

Structural equation modeling (SEM) was conducted in the IBM AMOS 24.0 software to test the conceptualized model. To confirm beyond any doubt the essential affiliation of the latent-structured model, a two-advance procedure constructed by [75] was used to test the conceptualized model and the mediating impact. To start with, the estimation model was used to test the confirmatory factor analysis (CFA); SEM was used to measure the path and fit coefficients of the conceptualized model. In accordance with [76], three item parcels were constructed for job satisfaction and CSEs with the factorial algorithm suggested by [77]. The following steps were followed to construct the item parcels: (1) the factor analysis of the item in each variable was conducted; (2) factor loadings were orchestrated in descending order; (3) all the items were assigned in turn to the three parcels according to their distinctive factor loading. This technique can obtain essentially identical factor loadings in each parcel. Moreover, AMOS user-defined stands were utilized to determine the particular and complete indirect effect together [78]. We utilized a bootstrapping investigation to measure the indirect impact. Bootstrapping strategies provide an exact estimation of the testing spread that is utilized to construct a certainty interval for indirect impacts [79].

## 4. Results

Table 1 provides the demographic characteristics of the sample of 305 academic staff, comprised of 80 (26.2%) females and 225 (73.8%) males. The participants ranged in age from 25 to 65 years and above; most participants (41.0%) were between the ages of 35–44. Most of the participants (192, 63.0%) had a PhD degree, while 230 participants (75.4%) had a job tenure of 5 years or more.

### 4.1. Measurement Model

In this research, the measurement model included five latent constructs: core self-evaluations, intrinsic motivation, extrinsic motivation, job satisfaction, and turnover intention, and 19 observed variables. Each dimension was tested for factor loadings, goodness of fit, convergent validity, squared different relationship, interdimensional relationship, discriminant legitimacy of the CFA model, and composite reliability. This test was subjected to normality checks; the kurtosis and skewness of the 19 items extended from −1.215 to 5.478 and from −1.947 to 0.690, respectively. Consequently, the items fit the presumptions of the confirmatory factor investigation for the test [80]. The primary test of the model measurement showed a very good fit to the data; the factor loadings were all significant (*p* < 0.001), suggesting that all the inactive constructs were well represented by their indicators, with the following acceptable fit statistics: CMIN = 226.375; DF = 142; CMIN/DF = 1.59; SRMR = 0.177; RMSEA = 0.044; GFI = 0.929; CFI = 0.966; TLI = 0.959; IFI = 0.966. As shown in Table 2, all estimation items stacked well on their individual components and surpassed 0.50, supporting the convergent validity of the measures [81].

To determine the discriminant legitimacy, the average variance extracted (AVE) of all sets of constructs reaching or surpassing 0.5 [79] was compared to the squared correlation of the other constructs. In this study, we compared the AVE square root with the Pearson’s relationship among measurements. As shown in Table 2, the AVE for each construct was more critical than the squared correlation of other constructs, illustrating support for discriminant legitimacy [81]. The data presented in Table 2 show that the composite reliability score was higher than 0.60 [80].

### 4.2. Correlational Analysis

The means, results from the correlational analysis, and standard deviations are displayed in Table 3. Core self-evaluations (CSEs) were positively connected to intrinsic motivation (IM) (*r* = 0.42, *p* < 0.05) and related to turnover intention (TI) (*r* = −0.259, *p* < 0.01), but not related to extrinsic motivation (EM) or job satisfaction (JS). There was a positive relation between intrinsic motivation and job satisfaction *(r* = 0.18, *p* < 0.01), and a negative association with turnover aims (*r* = −0.25, *p* < 0.01). There was no association between EM and JS, but there was a positive correlation with TI (*r* = 0.17, *p* < 0.01).

### 4.3. Tests of Hypothesized Model

**Figure 1:**
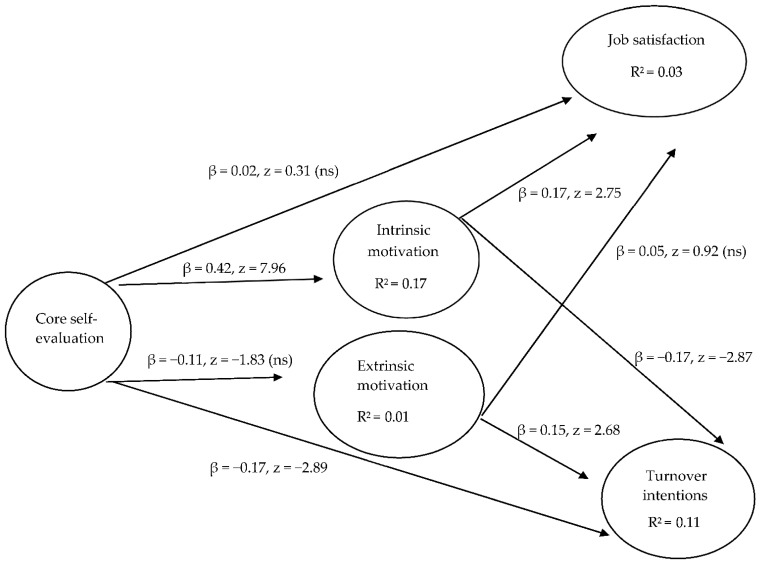
Hypothesized model: *Model fit statistics:* CMIN/DF = 0.021; CFI = 1.000; RMSEA = 0.000; SRMR = 0.062; GFI = 1.000; AGFI = 1.000; IFI = 1.019; and TLI = 1.101*. Notes:* All path estimates are significant except the direct path from core self-evaluations to job satisfaction and extrinsic motivation and the path from extrinsic motivation to job satisfaction. Not significant = ns; CMIN (Minimum discrepancy); CFI (Comparative fit index); RMSEA (Root mean square error of approximation); SRMR (Standardized root mean square residual); GFI (Goodness of fit); AGFI (Adjusted goodness of fit statistics); IFI (Incremental fit index); TLI (Tucker Lewis index).

The fit indicators (see Figure 1) show that the hypothesized model fit the data (CMIN/DF = 0.021; *p* < 0.979; CFI = 1.000; RMSEA = 0.000; SRMR = 0.062; GFI = 1.000; AGFI = 1.000; IFI = 1.019 and TLI = 1.101). Based on the findings, the path coefficients from CSEs to IM was positively significant (β = 0.42, z = 7.96, *p* < 0.001). Hence, H1a is empirically supported. However, the path coefficient from CSEs to EM was not significant (β = −11, z = −1.83, *p* < 0.07). Thus, H1b is not empirically supported. The findings showed that the path coefficient from CSEs to JS was not significant (β = 0.02, *z* = 0.31, *p* < 0.76). Thus, H2 is not supported. However, there is empirical support for H3; the path coefficient from CSEs to TI shows it was negatively significant (β = −0.17, z = −2.89, *p* < 0.01). In addition, the path coefficient from IM to JS was positively significant (β = 0.17, z = 2.75, *p* < 0.05) and that from EM to JS was not significant (β = 0.05, z = 0.92, *p* < 0.36). Thus, H4a is supported and H4b is not supported. Lastly, the path coefficient from IM to TI was negatively significant (β = −17, z = −2.87, *p* < 0.001) and that from EM to TI was positively significant (β = 0.15, z = 2.68, *p* < 0.05). Hence, H5a and H5b are supported.

Table 4 shows the findings of the parallel mediation effects of intrinsic and extrinsic motivation. A bootstrapping strategy that used 5000 test measures, a bias-corrected percentile, and a high-certainty interval was utilized to analyze the importance of different methods. The bootstrapped certainty interval did not incorporate zero [82].

As shown in Table 4, the parallel mediation (IM) fully mediated the effect of CSEs on JS (indirect effect = 0.077; LLCI = 0.013; ULCI = 0.155; *p* < 0.05) and EM fully mediated the effect of CSEs on JS (indirect effect = −0.005; LLCI = −0.035; ULCI = 0.005; *p* < 0.25) and did not include zero. Therefore, H6a and H6b are supported. IM partially mediated the effect of CSEs on TI (indirect effect = −0.033; LLCI = −0.070; ULCI = −0.004; *p* < 0.05) and EM partially mediated the effect of CSEs on TI (indirect effect = −0.007; LLCI = −0.025; ULCI = −0.002; *p* < 0.14) and did not include zero. Therefore, H7a and H7b are supported. The results explained 35% of the variance in CSEs, 13% of IM, 15% of EM, 49% of JS, and 9% of TI.

## 5. Discussion

The purpose of the research was to explore the relationship between CSEs, turnover intention, and job satisfaction among academic staff from HEIs in Nigeria using a parallel mediation of intrinsic/extrinsic motivation. The underpinning theory of self-determination theory [83] was used to test the hypothesized relationship. The findings of this research provided proof of the relationship between CSEs, job satisfaction, and turnover intention, while both intrinsic and extrinsic motivation mediated the relationship. Hypothesis 1a indicated that CSEs have a positive significant impact on intrinsic motivation and is strongly supported; the findings are in line with the tenets of self-determination theory [30]. Intrinsic motivation encourages performing with a sense of free will; when a person has a special interest in something, they will perform tasks with complete willingness [84]. According to [27], a person with greater CSEs recognizes their tasks as intrinsic attributes. The findings from the present study showed that CSEs increased job satisfaction through intrinsic motivation because intrinsic motivation encourages complete willingness to doing a job and thereby gaining satisfaction. This is consistent with principles of self-determination theory [80], showing the role of need fulfillment in gaining an understanding of the most noteworthy style of inspiration, and to perceive what motivates scholars to conduct research.

Hypothesis 1b suggesting that Core self-evaluations have a positive significant impact on extrinsic motivation was not supported in this study. It is often stated that a person can be motivated by extrinsic benefits, for example, promotion and compensation. Self-determination theory proposed that controlled motivation recommends that character can be different in situations where they are being controlled [25] which implies that use of extrinsic compensation may not increase motivation. However, the findings of this study dispute the notion that extrinsic compensation encourages extrinsic motivation to fulfill job satisfaction in academic staff, possibly because of the dissatisfaction of the financing of HEIs in Nigeria [85].

Hypothesis 2 states that core self-evaluations have a positive significant and indirect impact on job satisfaction; this research found that CSEs have no positive significant impact on job satisfaction, contrary to previous research that has shown a significant link between job satisfaction and personality characteristics [86] and that CSEs can positively anticipate job satisfaction [20,22]. Job satisfaction for HEIs academic staff in Nigeria is possibly more dependent on what they perceive as important in their jobs; if unable to obtain it, they become dissatisfied [9]. The academic staff in HEIs in Nigeria frequently complained that they are not adequately involved in decision making, which is a violation of their rights. Due to this reason, they are frustrated and dissatisfied which effects their commitment [85]. The constant strikes by Academic Staff Union of Universities (ASUU) shows the unfit HEIs system in Nigeria. The academic staff are dissatisfied with the work environment, financing of HEIs, and earned allocation. It is suggested that if staff are satisfied with their job, they will show significant commitment [85]. Normally, it is assumed that high-CSE persons may have higher satisfaction with their job than a person with lower CSEs because of the positivity and control they have towards their jobs [87] this may be another reason for the insignificant effect CSEs have on job satisfaction of academic staff in HEIs in Nigeria.

Hypothesis 3 indicating that core self-evaluations are negatively related to turnover intention is strongly supported. CSEs are negatively significant to turnover intention, which aligns with a previous research study that discovered that CSEs have a negative significant effect on turnover intention [19,23,43]. Individuals with lower CSEs may have less motivation, which in turn causes them to want to leave their organization.

Hypothesis 4a suggested that intrinsic motivation has a positive significant impact on job satisfaction; the results also support the positive effect of intrinsic motivation on job satisfaction, which is in line with the previous result reported in [53]. Hypothesis 4b indicating that extrinsic motivation has a positive significant impact on job satisfaction was not supported in the present study, contrary to several previous studies, but in line with the previous study that discovered extrinsic motivation has a negative impact on job satisfaction: “the negative sign for extrinsic motivation and job satisfaction is a surprise” an explanation is that this is a type of “crowding in” when processing intrinsic motivation can decrease extrinsic motivation [46].

Hypothesis 5a and Hypothesis 5b stating that intrinsic and extrinsic motivation have a negative significant impact on turnover intention were supported in this study, which aligns with a previous research study that discovered that intrinsic and extrinsic motivation had a negative impact on turnover intentions [23,65].

Hypothesis 6a and Hypothesis 6b suggesting that intrinsic and extrinsic motivation mediate the relationship between CSEs and job satisfaction are strongly supported, and Hypothesis 7a and Hypothesis 7b suggesting that the relationship between core self-evaluations and turnover intention is mediated by intrinsic and extrinsic motivation are also strongly supported because the parallel mediating role of intrinsic and extrinsic motivation gave new insight into the connection between CSEs, job satisfaction, and turnover intention. Earlier investigations found CSEs to be emphatically related to career commitment, staff compensation, and the tendency to urge for administrative changes [13,76]. However, in the current research, we built on earlier research and discovered that motivation (intrinsic and extrinsic) mediates the impact of CSEs on job satisfaction and turnover intentions.

In the parallel mediation model, the indirect link between CSEs and job satisfaction was mediated via both intrinsic and extrinsic motivation, which is like the findings of [38], who discovered a divergent link between CSEs and job satisfaction, mediated by perceived job characteristics. Hence, the discovery of the mediation impact of intrinsic and extrinsic motivation on the relationship between CSEs, job satisfaction and turnover intention may yield a few suggestions for the management of organizations. No study has employed intrinsic and extrinsic motivation as parallel mediators. The findings confirmed a connection between CSEs, motivation, job satisfaction, and turnover intention. Motivation leads to increased job satisfaction; this suggests how the traits of CSEs are applied. Additionally, to make advances regarding work fulfillment and to decrease turnover intention, CSEs might give a positive way of expanding motivation. Within the context of these discoveries, managers may improve staff emotions by providing a friendly and flexible work environment and selecting people who are more positive and certain in their thoughts (e.g., those who demonstrate self-esteem, enthusiasm, and stability) to improve their work fulfillment and decrease their intention to leave. In addition, of importance to human resource management by selecting people in the hiring process who score highly positive on the CSEs scale, they might view their work as more satisfying, consider some part of their job with higher regard, and develop more loyalty to their company for being satisfied [38].

The theoretical contribution in the present study is the first empirical work to explain the relationship between CSEs, intrinsic and extrinsic motivation, job satisfaction, and turnover intentions in a mediation model. As past study explored the relationship between the variables of this research separately and gave outcomes in parts; instead, this research suggested a combined perspective of the variables. Regarding the empirical proof regarding CSEs, job satisfaction, and turnover intention, we proposed and tested them using a mediation model; self-determination theory was used as the theoretical framework for the hypothesized model, hypothesizing the mediator role of intrinsic and extrinsic motivation in the relationship.

The present research significantly contributes to the existing literature on CSEs, job satisfaction, and turnover intentions by exploring the relationship introducing a mediator and using a sample from academic staff in HEIs. First, previous study showed that CSEs have a significant effect on job satisfaction [20,22] the present research discovered no direct effect but an indirect effect with a mediator. Hence, we used intrinsic and extrinsic motivation as a mediator. Second, only a few research works tested the link between CSEs, job satisfaction, and turnover intention and used different mediators such as perceived job characteristics [38], goal setting, and performance [64], but no study has used intrinsic and extrinsic motivation as parallel mediators. Third, not enough study has been conducted to investigate academic staff motivation in HEIs [6,88]. This is the first study to explore the impact of CSEs on job satisfaction and turnover intention through the parallel mediator of intrinsic and extrinsic motivation in academic staff in HEIs in Nigeria.

## 6. Limitations and Future Studies

A few limitations of the study need to be considered. First, a causal link between the research factors ought to be drawn with caution. Future investigations will utilize longitudinal strategies to confirm these connections. Second, the research depended on academic staff; future studies can obtain data from staff in other industries, such as banks, airlines, or healing centers, or from diverse districts such as Europe, Asia, or other African nations, which would be valuable in generalizing the associations determined. It is uncertain whether the study findings can be generalized to other job sectors. Third, future studies should examine other conceivable mediating factors (such as work engagement or career adaptability) within the relations between CSEs and work outcome. The existence of other causal relationship between CSEs and career adaptability needs to continue to be investigated in future studies [89].

Regardless of these limitations, the current study aimed to examine inspiration while at the same time considering how to expand our understanding of the components between CSEs, work fulfillment, and turnover intention. The results demonstrate that motivation purposefully mediates the relationship between CSEs, job satisfaction, and turnover. The study thus may give important assistance for how to implement interventions to advance the work output of scholarly staff.

## Figures and Tables

**Table 1:** Demographic characteristics.

	Frequency	%
Age		
25–34	37	12.1%
35–44	125	41.0%
45–54	89	29.2%
55–64	44	14.4%
65+	10	3.3%
Gender		
Female	80	26.2%
Male	225	73.8%
Education		
Undergraduate	10	3.3%
Masters	103	33.8%
Doctorate	192	63.0%
Job Tenure		
Less than 1 year	4	1.3%
From 1 to 3 years	25	8.2%
From 3 to 6 years	46	15.1%
6 years and above	230	75.4%

**Table 2:** Factor loadings and CFA results.

	Loadings	*t*-Value	AVR	CR	α
Core self-evaluations			0.51	0.76	0.71
CSES1	0.72	9.72			
CSES2	0.68	9.51			
CSES3	0.74	1.00			
Extrinsic motivation			0.60	0.84	0.89
EM1	0.70	11.08			
EM2	0.81	12.49			
EM3	0.81	12.58			
EM4	0.72	1.00			
Intrinsic motivation			0.52	0.86	0.85
IM1	0.56	8.01			
IM2	0.66	8.98			
IM3	0.71	9.46			
IM4	0.87	10.55			
IM5	0.88	10.65			
IM6	0.57	1.00			
Job satisfaction			0.44	0.70	0.70
JS1	0.73	6.67			
JS2	0.73	6.65			
JS3	0.51	1.00			
Turnover intention			0.76	0.90	0.90
TI1	0.93	22.89			
TI2	0.75	16.74			
TI3	0.92	1.00			

**Table 3:** Means, standard deviation, and correlations of all variables.

Variables	Mean	SD	CSEs	IM	EM	JS	TI
1. Core self-evaluations	42.14	6.02	-				
2. Intrinsic motivation	23.62	3.99	0.415 *	-			
3. Extrinsic motivation	12.51	3.98	−0.105	−0.38	-		
4. Job satisfaction	43.19	7.33	−0.084	−0.177 **	0.044	-	
5. Turnover intention	7.74	3.27	−0.259 **	−0.248 **	0.170 **	−0.029	-

*Notes:* SD—Standard deviation. * Correlation significant at the *p* < 0.05 level (one tailed); ** Correlation significant at the *p* < 0.01 level (two tailed).

**Table 4:** Bootstrapping findings of the mediating effects of intrinsic and extrinsic motivation.

Hypothesized Mediating Relationships	Unstandardized Indirect Estimates	LLCI	ULCI	*p* < 0.05
Core self-evaluations–intrinsic motivation–job satisfaction (0.28 × 0.28)	0.077	0.013	0.155	0.05
Core self-evaluations–extrinsic motivation–job satisfaction (−0.06 × 0.09)	−0.005	−0.035	0.005	0.25
Core self-evaluations–intrinsic motivation–turnover intention (0.28 × −0.12)	−0.033	−0.070	−0.004	0.05
Core self-evaluations–extrinsic motivation–turnover intention (−0.06 × 0.11)	−0.007	−0.025	0.002	0.14

*Notes:* 5000 samples generated from a 90% confidence interval (CI) were received to test the meaning of the aberrant impacts using the bootstrapping method. Gender, age, job tenure, and education were the control variables. LLCI (lower-level confidence intervals); ULCI (upper-level confidence intervals).

## Data Availability

Data are not publicly available but may be made available upon request from the corresponding author.
